# Long-Term Outcomes of Inverted-Bearing Reverse Shoulder Arthroplasty with Large Polyethylene Glenospheres

**DOI:** 10.3390/jcm15176533

**Published:** 2026-08-24

**Authors:** William G. Blakeney, David Graham, Stefan Bauer, Peter Campbell

**Affiliations:** 1 West Coast Orthopaedic Centre, Subiaco, WA 6008, Australia; 2 Department of Orthopaedic Surgery, Royal Perth Hospital, Wellington Street, Perth, WA 6000, Australia; 3 Department of Surgery, The University of Western Australia, Perth, WA 6009, Australia; 4 Ensemble Hospitalier de la Côte, Chirurgie de l’Épaule, CH 1110 Morges, Switzerland

**Keywords:** reverse total shoulder arthroplasty, inverted bearing, polyethylene glenosphere, scapular notching, humeral stress shielding

## Abstract

**Background**: Inverted-bearing reverse total shoulder arthroplasty (rTSA), using a polyethylene glenosphere with a metal humeral liner, may reduce polyethylene wear, osteolysis, and scapular notching. This study evaluated long-term survivorship, complications, radiographic findings, and clinical outcomes of a large-glenosphere inverted-bearing rTSA design. **Methods**: A retrospective single-surgeon cohort included 394 primary rTSAs (363 patients; mean age 73.8 ± 8.5 years) performed between 2006 and 2018 using the same implant system. Clinical follow-up was available for 123 patients at a mean of 9.4 years. Outcomes included ASES, Oxford Shoulder Score (OSS), Subjective Shoulder Value (SSV), and pain VAS. Radiographs obtained at ≥8 years were assessed for scapular notching, glenoid loosening, and humeral osteolysis/stress shielding. Kaplan–Meier survival analysis was performed. **Results**: Thirty of the 257 rTSAs with revision follow-up (11.7%) underwent revision, most commonly for glenoid loosening or instability. Among patients with long-term radiographs, scapular notching occurred in 8.3% of all-polyethylene glenospheres and was limited to grade 1. Humeral osteolysis/stress shielding was present in 36%, although no humeral components were revised for loosening. Median final follow-up PROMs were ASES 75.8, OSS 42, SSV 78, and pain VAS 1. **Conclusions**: Large-glenosphere inverted-bearing rTSA demonstrated satisfactory long-term outcomes, low notching rates, and acceptable survivorship. The favourable notching profile should be interpreted in the context of the larger eccentric glenosphere design, which precludes attribution of this finding to bearing material alone.

## 1. Introduction

Reverse total shoulder arthroplasty (rTSA) has evolved significantly since its inception, expanding its indications beyond rotator cuff tear arthropathy to include many complex shoulder conditions [1,2,3,4,5]. Long-term studies have demonstrated sustained improvements in shoulder function, patient satisfaction and pain relief following rTSA [6,7,8,9,10].

One of the prevalent concerns in rTSA is the occurrence of scapular notching [11,12,13,14,15], a notable complication that has adverse effects on shoulder function [14] and can lead to loosening [15]. Notching was classified by Sirveaux et al. into grades 1–4 [16]. Early notching (grades 1 and 2) is a result of mechanical impingement of the humeral liner against the scapular pillar. Initially, adduction was believed to be the main cause [17]. However, it is more likely a result of internal and external rotation with the arm at the side [18] or “friction-type impingement” [19]. Progression to more advanced notching (grades 3 and 4) is secondary to polyethylene debris from mechanical wear leading to a chronic inflammatory response and osteolysis [17].

Over recent years, attention has turned towards methods to avoid notching. The main focus has been on implant positioning to avoid mechanical impingement: lateralization of the glenosphere and humerus [20] and inferiorizing and posteriorizing the glenosphere [21]. Increasing glenosphere size is another alternative [22]. Inverted-bearing rTSA involves a polyethylene glenosphere articulating with a metallic humeral liner, theoretically reducing the volume of polyethylene wear debris, improving resistance to mechanical degradation, and minimizing osteolysis and associated complications.

Mid- to long-term follow-up studies have shown promising results [23,24]. A multicentre study reported significant improvements in Constant scores and range of motion at a mean follow-up of 7 years, with a low incidence of high-grade scapular notching [23]. A single-centre study with a mean of 10 years of follow-up similarly demonstrated good functional outcomes with a 98.7% survivorship. Scapular notching was reported in 51.4% of patients, but these were almost entirely low grade (only one patient had grade 3 notching).

The primary aims of this study were to report survivorship, complications and radiological outcomes (focusing on occurrence of scapular notching and humeral stress shielding) of a large glenosphere, inverse-bearing rTSA at long-term follow-up. The secondary endpoint was to report clinical outcomes.

## 2. Materials and Methods

This was a single surgeon retrospective study of a consecutive series of 394 RSAs in 363 patients (226 females (62.3%), 137 males (37.7%)) who underwent primary reverse shoulder arthroplasty between January 2006 and June 2018. The diagnosis at time of surgery is presented in Table 1. The mean age at surgery was 73.8 years (SD 8.5).

**Figure 1:**
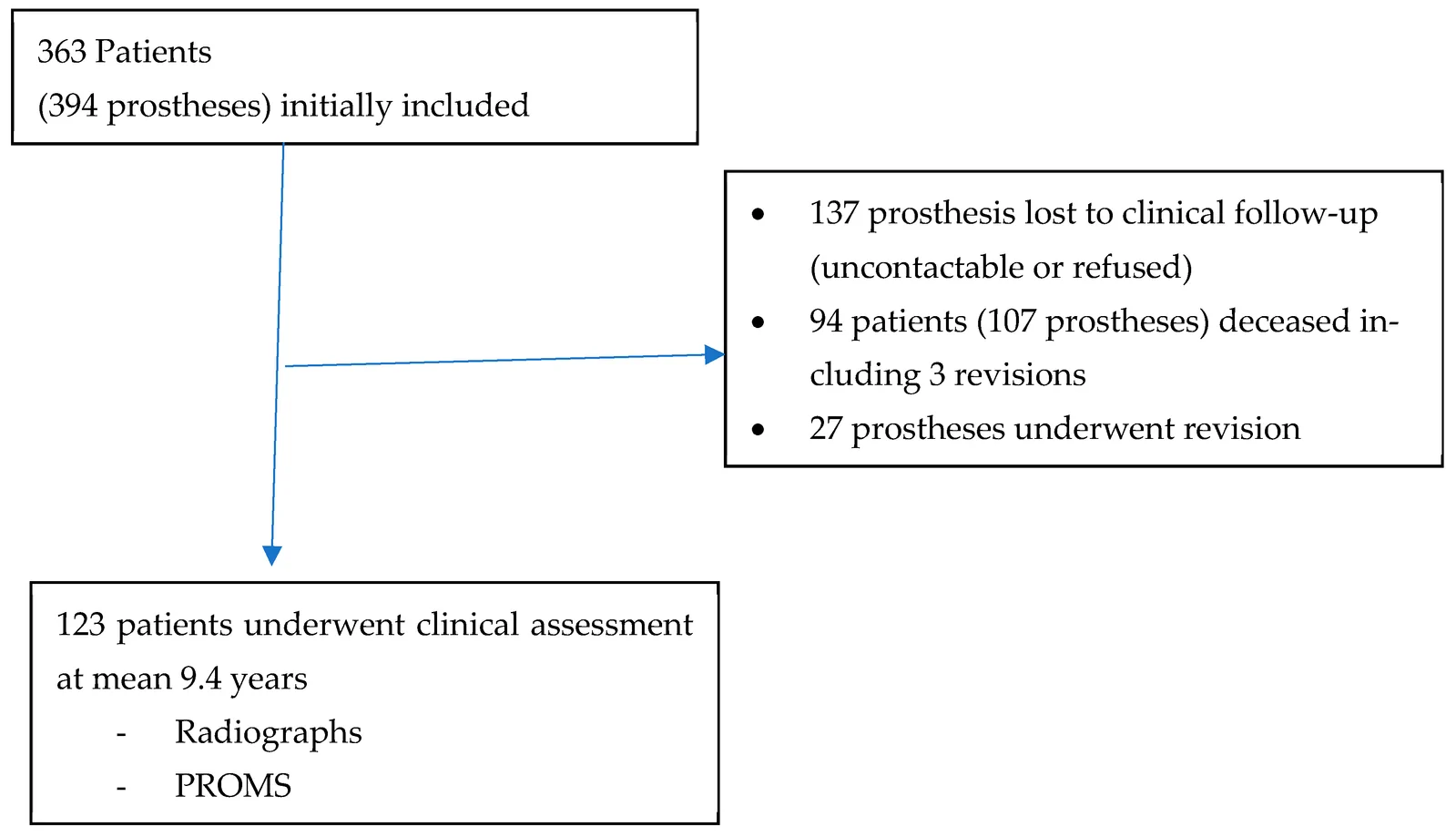
Flow chart of study patients.

Figure 1 summarises patient flow in the study. A total of 137 rTSAs (34.7%) were lost to follow-up (uncontactable or refused follow-up). Another 94 patients with 107 rTSAs were deceased during the study period. Three of these rTSAs had undergone revision prior to death. A further 27 rTSAs underwent revision surgery (total of 30). The remaining 123 patients underwent clinical assessment at a mean of 9.4 years post-surgery (range: 6.7–16.6 years). The following patient-reported outcome measures (PROMs) were recorded: American Shoulder and Elbow Society Score (ASES), Oxford Shoulder Score (OSS) and the Subjective Shoulder Value (SSV). Patients completed these electronically or were sent a paper version if not computer literate.

All patients had the same reverse prosthesis SMR (Lima Corporate, Udine, Italy) performed using the same technique, through a delto-pectoral approach. The SMR inlay cementless stem with a 150° neck-shaft angle was used for all patients. The SMR L1 base plate was used in 334 cases and the L2 base plate was used in 60 cases. The glenosphere used was an all-polyethylene 44 mm eccentric in 347 patients, all-polyethylene 40 mm concentric in 9 patients and metallic 36 mm eccentric in 38 patients. The metallic glenosphere represents the conventional-bearing configuration for this implant system, whereas the polyethylene glenosphere represents the inverted-bearing configuration. The subscapularis tendon was not repaired in any patients.

Patients had digital radiography performed at a minimum of 8 years post-surgery. Eight patients were unavailable for radiography, leaving 116 patients. Radiographs were assessed independently by 2 experienced shoulder surgeons and an experienced musculoskeletal radiologist. Radiographs were assessed for implant failure or loosening, notching (Sirveaux classification) or areas of radiolucency in the glenoid and osteolysis or stress shielding in the humerus, with areas of radiolucency around the humeral stem recorded from zone 1 laterally to zone 7 medially, with zone 4 being around the tip of the stem. Notching grade and humeral radiolucency severity were scored by the 2 experienced shoulder surgeons.

### Statistics

Data were reported using medians and interquartile ranges due to the skewed distributions and analysed using Mann–Whitney U tests. Categorical data were reported as frequencies and percentages and analysed using Fisher’s exact test. The time to revisions was analysed using survival analysis, presented using Kaplan–Meier curves and analysed using a Cox proportional hazards model. Survival analysis censored deaths and loss to follow-up at last contact. Interobserver agreement was evaluated using weighted Cohen’s kappa statistics. Kappa values were interpreted according to Landis and Koch criteria (≤0.20 slight, 0.21–0.40 fair, 0.41–0.60 moderate, 0.61–0.80 substantial, and >0.80 almost perfect agreement). Statistical significance was set at *p* < 0.05. All statistical analysis was performed using Stata v 16.1 (StataCorp, College Station, TX, USA). For patient-reported outcome measures (PROMs), Tobit regression using a mixed-effects model with patient as a random effect was performed to account for the non-independence of bilateral shoulder arthroplasties. Tobit regression was selected because ceiling effects were observed across all PROMs. Alternative modelling approaches, including gamma regression and data transformation, were explored but did not materially alter the results.

## 3. Results

**Figure 2:**
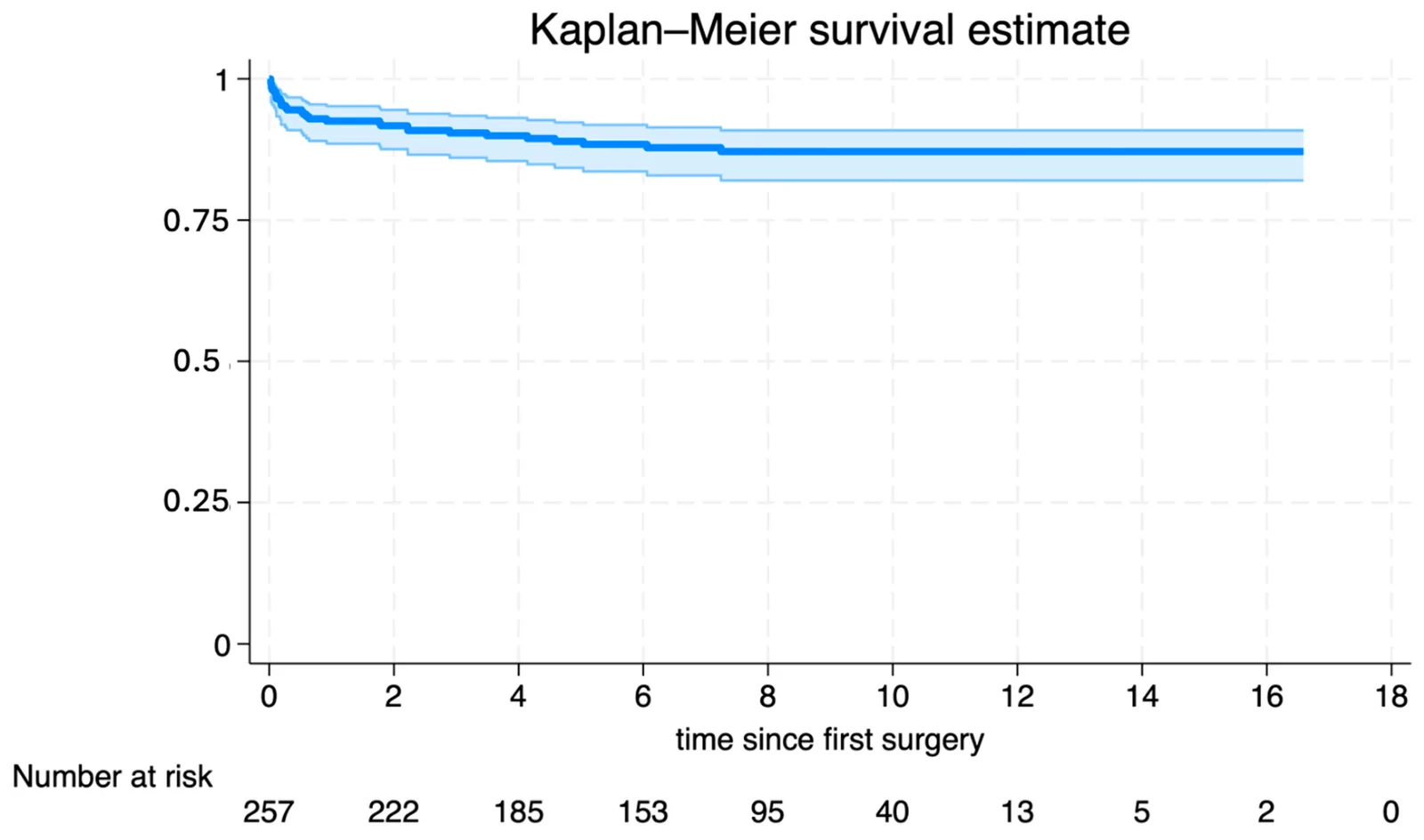
Kaplan–Meier survivorship chart.

The Kaplan–Meier survival estimate of the 257 rTSAs not lost to follow-up is demonstrated in Figure 2. Implant survivorship was 89% at 5 years (95% C.I. 84–92) and 87% at 10 years (95% C.I. 82–91).

### 3.1. Revisions

Thirty of the 257 rTSAs not lost to follow-up (11.7%) underwent revision surgery. Most of the revisions were early (median 0.5 years, IQR 0.2–1.8). Of these, 10 patients had glenoid loosening, 10 patients were revised for instability and four patients were revised for infection. Two patients had a stem revision for a periprosthetic proximal humerus fracture and two patients underwent open reduction internal fixation for a scapula spine fracture.

### 3.2. Radiological Evaluation

**Figure 3:**
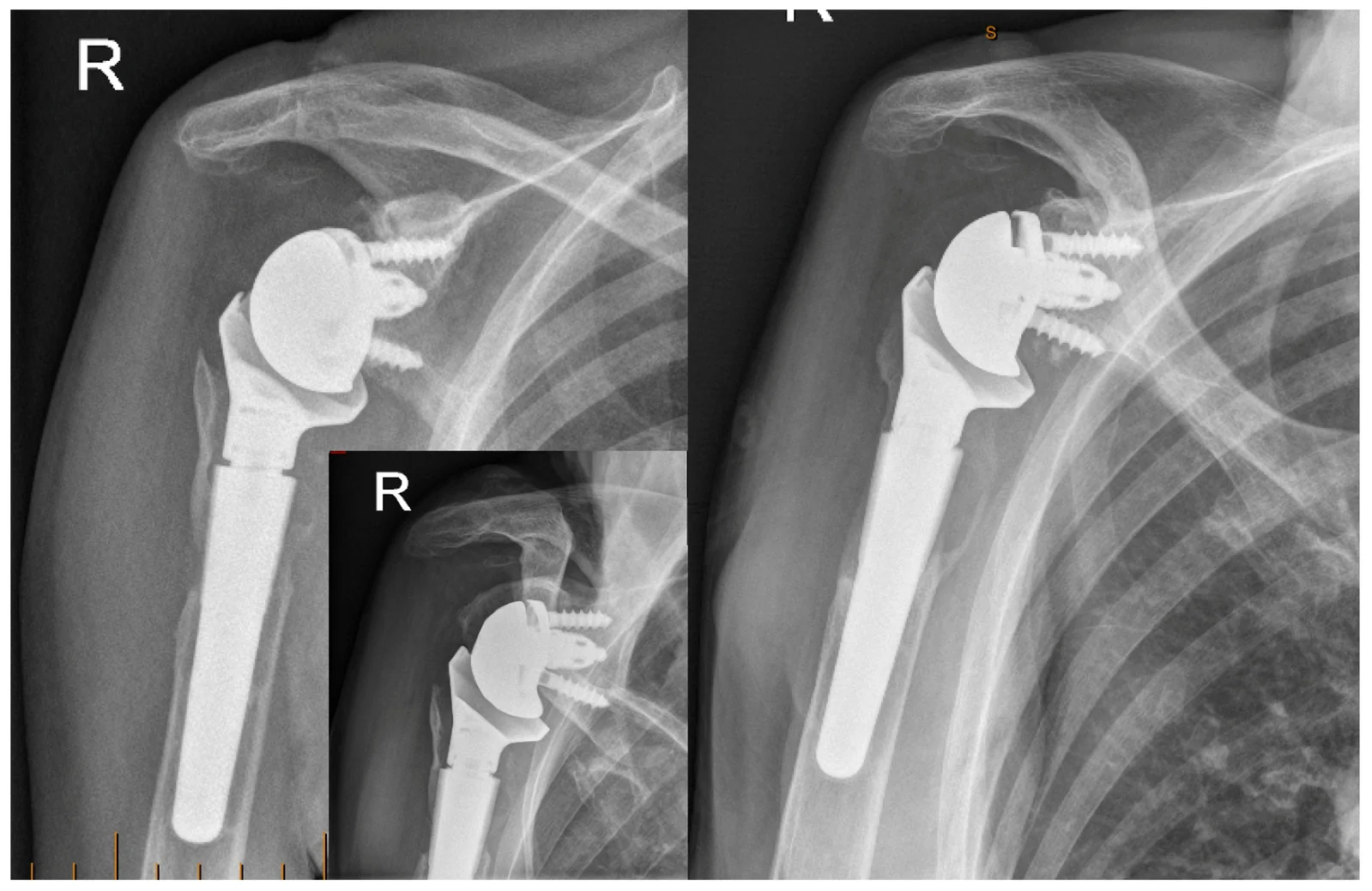
Radiograph of patient with a metal glenosphere taken at 10 and 16 years postoperatively. Note notching progressing to grade 3, with progression of stress shielding around humeral component.

**Figure 4:**
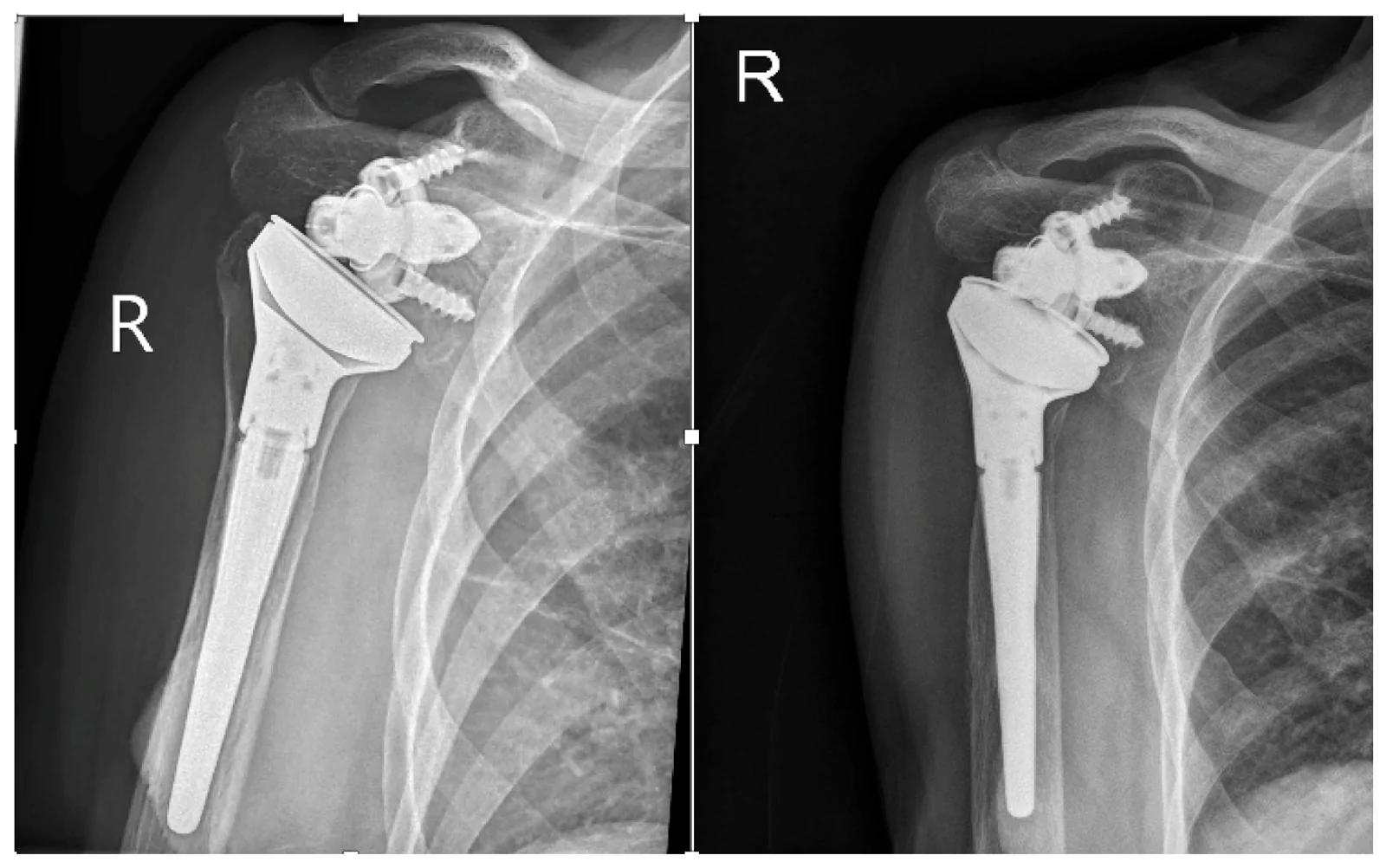
Radiograph of patient with a polyethylene glenosphere taken at 8 and 14 years postoperatively. Note grade 1 notching progressing to grade 2, with progression of stress shielding around humeral component.

Of the 109 rTSAs with a 40 or 44 mm all-polyethylene glenosphere, scapular notching was present in nine (8.3%) patients (all grade 1). No cases of grades 2, 3 and 4 were observed. Of the seven patients that had a metal glenosphere, three (43%) had notching (one grade 1, one grade 2 and one grade 3). This difference in notching rates between metal and polyethylene glenoids was significant (*p* = 0.024). However, given the very small number of patients with long-term radiographic follow-up in the metal glenosphere group, this comparison should be interpreted with caution. Figure 3 and Figure 4 demonstrate notching and stress shielding seen at long-term follow-up.

Weighted Cohen’s kappa demonstrated almost perfect interobserver agreement for both Sirveaux notching (κ = 0.88) and humeral radiolucency/stress shielding (κ = 0.84).

Humeral radiolucency/osteolysis secondary to stress shielding was seen in 42 patients (36%). Twelve cases had osteolysis in one zone, 19 in two zones, eight in three zones and three in four zones. The typical pattern seen starts in zone 1 and then 7, extending into zones 2 and 6 in more severe cases. It is associated with cortical thinning. Seventy-four patients (64%) had no osteolysis.

### 3.3. Clinical Evaluation

Patient-reported outcome measures at final follow-up are presented in Table 2. Of the 123 patients included in the PROM analysis, 115 (93.5%) contributed a single shoulder, while eight patients (6.5%) contributed bilateral reverse shoulder arthroplasties.

## 4. Discussion

This long-term single-surgeon series of inverted-bearing reverse total shoulder arthroplasty (rTSA) demonstrates durable functional outcomes, low rates of high-grade scapular notching, and a favourable implant survivorship profile at a mean of 9.4 years postoperatively. These results provide further support for the theoretical advantages of large polyethylene glenospheres in minimizing polyethylene wear-related complications while maintaining excellent clinical efficacy.

The median postoperative ASES score of 75.8, OSS of 42, and SSV of 78 observed in our cohort are consistent with or superior to previous reports on long-term outcomes in rTSA populations, including those using conventional-bearing constructs [25,26]. Importantly, these outcomes were achieved in a relatively elderly cohort (mean age 73.8 years) with a broad range of underlying pathology, emphasizing the versatility and durability of the inverted-bearing design in real-world clinical practice.

A key finding of this study is the low rate of scapular notching in patients receiving all-polyethylene glenospheres, only 8.3%, and exclusively grade 1 notching. No cases of high-grade notching (grade ≥2) were observed in this group, which supports the hypothesis that inverted-bearing configurations can mitigate polyethylene impingement debris and its osteolytic sequelae. These findings are consistent with those of previous mid- to long-term series that have suggested reduced notching with polyethylene glenoid constructs [23,24]. In contrast, the metal glenosphere subgroup demonstrated a significantly higher notching rate of 43%, including one case each of grade 2 and 3 notching. However, only seven patients with metal glenospheres were available for long-term radiographic follow-up, and this comparison should therefore be regarded as exploratory rather than definitive.

Irlenbusch et al. evaluated the clinical and radiographic results of rTSA with inverted-bearing materials over a two-year follow-up period, noting promising trends in patient outcomes [27]. Inferior scapular notching (only grades 1 and 2) was identified in 20.5% of patients. These findings were replicated in a minimum 2-year follow-up study by Paccot et al. with high patient-reported and functional outcome scores, and grade 1 notching present in 33% and grade 2 in 5%. Both studies reported no signs of grade 3 or 4 notching. The lower notching rates seen in the present study may be explained by the large number of 44 mm eccentric glenospheres used in this cohort (328 of 337, 97.3%). All of the polyethylene glenosphere patients available for radiographic follow-up had a 44 eccentric glenosphere. Using a large eccentric glenosphere will increase the lateral and inferior offset of the glenosphere from the scapular pillar, increasing impingement-free range of motion regardless of bearing surfaces [21,22,28]. This finding is particularly noteworthy given that all patients received a 150° inlay humeral stem, a design traditionally associated with an increased risk of scapular notching compared with more lateralized or lower neck-shaft-angle designs [29]. A retrospective study by Poon et al. of 145 SMR rTSAs comparing the larger polyethylene glenospheres to smaller metal ones, showed similar rates and severity of scapular notching with the polyethylene group. At a minimum of 2 years of follow-up, notching was noted in 16 (25%) of the metal glenospheres vs. nine (11.1%) of the polyethylene glenospheres (*p* = 0.028). Functional outcomes (Oxford Shoulder Score) were higher and complication rates lower in the polyethylene group, with no implant failures noted. This is in contrast with a study by Bacle et al. reporting high rates of notching (73%) at a minimum of 10 years of follow-up in rTSA with metal glenospheres [25].

The rate and pattern of humeral osteolysis observed—36% overall—reflect a common concern associated with uncemented press-fit stem designs [30,31]. The radiolucency most commonly involved zones 1 and 7 and extended to zones 2 and 6 in more severe cases, consistent with previously reported patterns of stress shielding and proximal bone resorption. Long (>100 mm) diaphyseal fit stems such as those used in the present study have been reported as a risk factor for stress shielding in a number of other studies [32,33,34], which accounts for the move towards smaller metaphyseal-fit stems in modern implants. While the clinical consequences of this radiographic phenomenon remain unclear, the presence of osteolysis in up to four zones in a minority of patients may warrant consideration in long-term monitoring and future revision planning. Importantly, no patients in this series were revised for humeral stem loosening. Although stress shielding appeared to progress radiographically in some patients over time (Figure 3 and Figure 4), no association with clinical symptoms or humeral component revision was identified. Castagna et al. reported similar rates of radiolucent lines secondary to stress shielding in a series of 126 rTSA at a mean of 7.2 years of follow-up with no revisions performed for humeral component loosening. Contemporary rTSA systems increasingly favour shorter, metaphyseal-fit stems to reduce stress shielding and simplify revision. While no humeral revisions for loosening occurred in this cohort, translation of these radiographic findings to modern metaphyseal-fit designs should be done cautiously; future work correlating stress-shielding severity with symptoms and revision risk remains warranted.

Revision surgery was required in 11.7% of patients, with the majority occurring within the first two years. The most frequent indications for revision were glenoid loosening and instability, each accounting for 33% of all revisions. The early timing of most revisions (median 0.5 years) suggests that failure modes were more commonly related to technical or early mechanical factors rather than late biological degradation or wear-related phenomena. The subscapularis tendon was not repaired in this series and may have contributed to some cases of instability. However, the effect of subscapularis repair on postoperative stability remains controversial. In the 10 cases of glenoid loosening recorded, these were early complications in elderly patients with osteoporotic bone. These were all early in the surgeon’s adoption of this implant. Alteration of the surgical technique, with judicious reaming to subchondral bone and augmentation of the screws with cement in osteoporotic patients, eliminated this complication.

This study is strengthened by its large cohort size, standardized surgical technique, consistent implant selection, and extended follow-up. The inclusion of radiographic assessment by both experienced surgeons and a musculoskeletal radiologist adds robustness to the interpretation of imaging findings. The study is also limited by substantial attrition, with 34.7% of shoulders unavailable for long-term clinical follow-up, which may introduce selection bias. However, high attrition is common in long-term reverse shoulder arthroplasty studies. A recent systematic review of studies with a minimum follow-up of 10 years reported loss to follow-up ranging from 47% to 80% (mean 63.3%), yet all included studies reported Kaplan–Meier survivorship, with estimates ranging from 73% to 94% (mean 87.9%) [35]. Therefore, while our survivorship estimates should be interpreted with appropriate caution, the methodology is consistent with contemporary long-term arthroplasty literature. Because all patients remained captured within the Australian Orthopaedic Association National Joint Replacement Registry, revision procedures performed elsewhere in Australia would still have been identified, making it unlikely that loss to follow-up materially influenced the reported implant survivorship. Although a subset of patients received conventional (non-inverted) metal glenospheres, enabling some level of comparison, the small sample size with extended radiographic follow-up in this group limits the strength of any conclusions regarding comparative effectiveness. Furthermore, the glenosphere size may act as a confounder, as with this implant design, the metallic glenosphere size is 36 mm compared to a 44 mm polyethylene glenosphere size. The low numbers of grade 1 and 2 notching cases in the polyethylene cohort suggests that perhaps simply using a large eccentric glenosphere is an effective way to reduce notching regardless of bearing surface. Finally, preoperative patient-reported outcome measures were not collected consistently and therefore changes in PROMs could not be assessed. In addition, standardized postoperative range-of-motion measurements were not uniformly available, limiting objective assessment of functional improvement.

## 5. Conclusions

Inverted-bearing reverse total shoulder arthroplasty using a large polyethylene glenosphere and metal humeral liner demonstrated satisfactory clinical outcomes, high implant survivorship, and low rates of scapular notching at a mean follow-up of nearly 10 years. The relatively low incidence of notching and glenoid loosening observed in this cohort may support some of the proposed biomechanical advantages of this bearing configuration. The favourable notching profile should be interpreted in the context of the larger eccentric glenosphere design, which precludes attribution of this finding to bearing material alone. However, humeral osteolysis was frequently identified radiographically, and the long-term clinical significance of this finding remains uncertain despite no observed increase in revision risk during the follow-up period. While these results suggest that the large glenosphere inverted-bearing design is a viable option in primary rTSA, further comparative studies and longer-term registry data are needed to better define its long-term performance.

## Figures and Tables

**Table 1:** Diagnosis at time of surgery.

Diagnosis	n = 394
Osteoarthritis	152
Cuff tear arthropathy	135
Massive irreparable cuff tear	62
Inflammatory arthritis	12
Acute fracture	12
Fracture sequelae	12
AVN	9

**Table 2:** Patient-reported outcome measures (PROMs) at final follow-up: American Shoulder and Elbow Society Score (ASES), activities of daily living, Subjective Shoulder Value (SSV), Visual Analog Score (VAS), and Oxford Shoulder Score (OSS). Note—Pain is per person. If a person has bilateral rTSAs then they are only given one pain score.

	n	Median (IQR)
SSV	122	78 (60, 90)
ASES	123	75.8 (58.3, 91.7)
ASES—ADLs	123	22 (15, 27)
Pain VAS	115	1 (0, 5)
OSS	123	42 (31, 46)

## Data Availability

The data presented in this study are available on request from the corresponding author.
